# Podocin and Beta Dystroglycan expression to study Podocyte-Podocyte and basement membrane matrix connections in adult protienuric states

**DOI:** 10.1186/1746-1596-9-40

**Published:** 2014-02-21

**Authors:** Praveen B Shankar, Ritambhra Nada, Kusum Joshi, Ashwani Kumar, Charan Singh Rayat, Vinay Sakhuja

**Affiliations:** 1 Department of Histopathology, Post Graduate Institute of Medical Education and Research, Chandigarh 160012, India; 2 Department of Nephrology, Post Graduate Institute of Medical Education and Research, Chandigarh 160012, India

**Keywords:** Beta dystroglycan, Focal segmental glomerulosclerosis, IgA nephropathy, Membranous glomerulonephritis, Minimal change disease, Podocin

## Abstract

**Background:**

Podocytes can be the primary site of injury or secondarily involved in various protienuric states. Cross talk between adjacent foot processes and with basement membrane is important for slit diaphragm function. Does expression of podocyte associated proteins in kidney biopsies alter with site/type of primary injury? Genetic mutations of podocin result in steroid resistant FSGS. Can protein expression of podocin predict resistant cases to initiate further genetic evaluation?

**Methods:**

Adult patients (n-88) with protienuria- minimal change disease(MCD)-22, focal segmental glomerulosclerosis(FSGS)-21,membranous glomerulonephritis(MGN)-25 and IgA nephropathy(IgAN)-20 were selected for immunohistochemistry with podocin and beta dystroglycan . Results were graded (0 - 3+scale )and compared with control biopsies and internal control. Treatment and follow up (6 months -2 ½ years) of FSGS and MCD cases were collected.

**Results:**

There was intense to moderate staining of the podocytes with podocin and β dystroglycan in the glomeruli in all cases (MCD, FSGS, IgAN and MGN) except for weak staining with β dystroglycan in 3 cases of MCD. There was loss of immunostains in areas of segmental/global sclerosis. There was no significant difference in the staining pattern between the groups. In primary podocytopathies, staining pattern did not differ between steroid resistant, sensitive or dependent cases.

**Conclusions:**

Immunohistochemical expression of podocin and β dystroglycan does not differ in nephropathies which have different site of injury depending on absence (MCD and FSGS) or presence of immune deposits and their localization (MGN and IgAN). Podocin and β dystroglycan staining did not differentiate steroid sensitive and resistant cases, hence, does not give clue to initiate genetic studies. However, analysis of bigger cohort may be required.

**Summary:**

Podocin and β dystroglycan immunohistochemistry was done to analyze podocyte - podocyte and podocyte -basement membrane matrix connections in adult protienuric states. Primary podocytopathies i.e. MCD and FSGS and secondary podocytopathy due to immune complex deposition i.e. MGN (subepithelial) and IgAN (mesangial) were analyzed. There was no difference in staining patterns between primary and secondary podocytopathies or between steroid sensitive, resistant and dependent cases of FSGS and MCD.

**Virtual slides:**

The virtual slide(s) for this article can be found here: http://www.diagnosticpathology.diagnomx.eu/vs/2258608781052786

## Background

Podocyte are important component of glomerular filtration barrier. There are primary podocytopathies where podocytes are aetiopathogenetically associated with the pathology i.e. MCD and FSGS. Podocytes can be secondarily involved and contribute to protienuria in diseases where primary site of injury is not podocyte e.g. membranous with sub epithelial immune complex deposits and IgAN with dominant mesangial immune deposits [1,2]. Podocyturia as a marker of disease activity has been reported in various protienuric states including FSGS, IgAN and MGN using various podocyte specific proteins [3] but not in MCD [4]. However, integrins including α and β dystroglycan which determine the attachment of podocytes to basement membranes have been shown to be reduced in steroid sensitive MCD and suggested to be useful in distinguishing FSGS, MCD and unrepresented FSGS [5-8]. Podocin being specific marker to identify podocytes in studies conducted for podocyturia, it might be worthwhile to study its expression in kidney biopsy in these protienuric states.

Various other podocyte associated proteins are being studied in these protienuric states using different methods yielding variable results [5-12]. Previous studies have been conducted in relatively smaller cohorts and composition of the cohorts in most of these studies were heterogeneous, comprising of both pediatric and adult patients; it is a known fact that aetiopathogenesis and clinical progression of nephropathies is different in adults and children. To explore answers to these questions we conducted this study on a cohort of 80 adult patients.

Podocin is one of the slit diaphragm proteins and mutations of its genes have been associated with non Finnish type of congenital and infantile nephrotic syndrome [13-16] and as steroid resistant nephrotic syndrome in relatively older siblings (up to 7 years) in such families [13]. Renal morphology is such cases can minimal glomerular changes or FSGS [13]. Mutations of genes for podocin have been documented even in sporadic cases of FSGS both in pediatric (10-30%) [14,15] and adult cohorts (<1% -12%) [17-20]. Steroids and cytotoxic therapies in such patients should be avoided as they are not expected to show any response. Another clinical implication is that FSGS with genetic abnormalities do not recur after transplant. Different type of mutations ranging from nonsense, missense and frame shift mutations have been reported which on functional analysis, in certain situations affect podocin-nephrin binding also [17-20]. Is there any difference in expression of podocin protein in these patients with genetic basis of FSGS and other protienuric states which can be identified by simpler test like immunohistochemistry specific for the podocin to identify candidates for further genetic studies?

This study was done to find whether podocin and beta dystroglycan protein expression differs in podocytopathies (primary and secondary- immune complex induced) in adults and will they help to differentiate between the two primary podocytopathies i.e. MCD and FSGS. Can pattern of expression of these proteins indicate steroid resistant non familial nephrotic syndrome? Whether these markers can predict response to steroid therapy? In protienuric conditions with podocyturia, is there altered expression of integrins i.e. beta dystroglycan, the adhesion molecules between podocytes and basement membrane.

### Subjects and methods

Eighty eight consecutive cases of adult patients who presented with nephrotic/subnephrotic range protienuria with (FSGS-21, IgAN-20) or without nephritic picture (MCD-22, MGN-25) between 1.1.2009- 30.9.2012 were chosen from the files of department of Histopathology, PGIMER Chandigarh. Thin needle core like tissue from nephrectomy specimens done for renal tumors and zero hour transplant biopsies were used as control with each batch of slides stained (each batch n-10). The clinical details and investigations were retrieved from the files of department of Nephrology, PGIMER. All the biopsies were analyzed by light microscopy, immunofluorescence and electron microscopy by two nephropathologist (KJ and RN). Only FSGS-NOS variant were included in this study. Since we intended to study podocytes in mesangial disease, cases of IgAN preferably with only/dominant mesangial deposits were included.

Immunohistochemistry for podocin and beta dystroglycan was performed using Rabbit Anti-human monoclonal IgG antibody. Antigen retrieval was done by heat retrieval under pressure using pressure cooker method. Primary antibody for Beta dystroglycan (Cat no.VP-B205, Vector laboratory) was used at a concentration of 1:30, while podocin (Cat podo 11-A Alpha diagnostic laboratory) was used at a dilution of 1:50. The secondary antibody tagged with peroxidase was used and color was developed with DAB. One control slide was stained with each batch.

**Figure 1:**
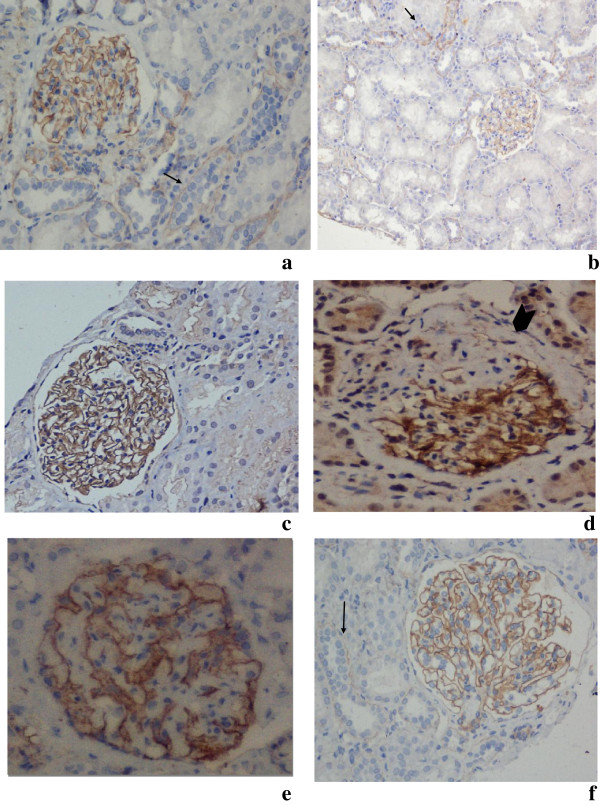
Photomicrograph shows beta dystroglycan expression (2+) in the glomeruli & tubules (arrows) a) Control (20X) b) MCD (10X) c) FSGS-normal glomerulus (20X) d) FSGS-loss of staining in sclerosed area (arrow head) (X40) e) IgAN (X40) f) MGN (20X) IHC, beta dystroglycan antibody.

**Figure 2:**
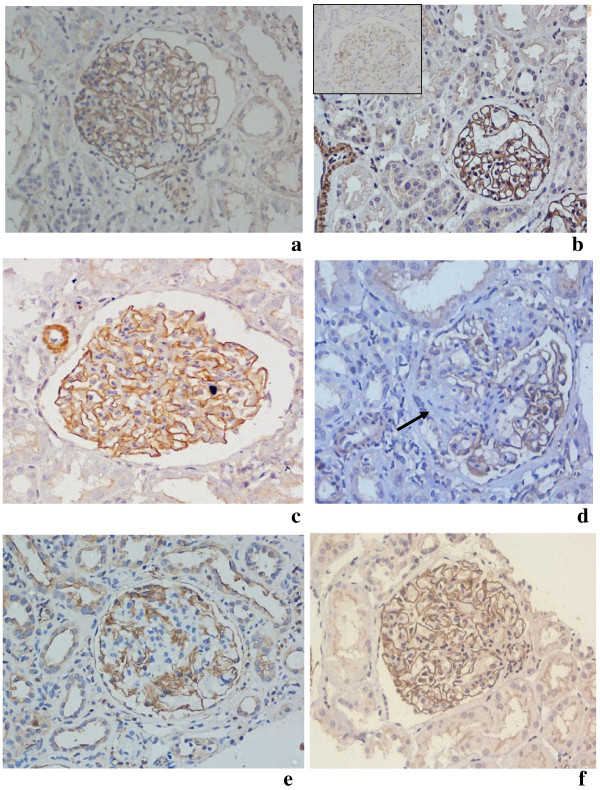
Photomicrograph shows podocin expression (2+) in all except 1+ intensity in MCD (inset b) and loss of staining in FSGS (d) a) control b) MCD c) & d) FSGS e) IgAN f) MGN) IHC, Podocin, X20.

Immunohistochemical staining in the glomeruli was compared with control biopsies and/or internal control, and graded on a scale of 0 to 3+. Control cases showed 2+ staining of podocytes and of distal tubular basement membrane with β-dystroglycan (Figure 1a). With podocin glomeruli and smooth muscles cells of blood vessels showed 2+ positivity with podocin (Figure 2a). Any global or segmental loss of expression of these podocyte proteins was noted, and the number of glomeruli affected was recorded. Staining in tubular basement membrane also served as internal control for immunohistochemistry for β-dystroglycan. 0 and 1+ were considered to be negative staining while 2+ and 3+ were considered to be positive staining.

Treatment and follow up history (6 months-2 ½ years) of FSGS and MCD cases was collected. They were classified as steroid sensitive, steroid resistant or steroid dependent groups. Follow up history was available in ten cases of MCD and twelve cases of FSGS. Of these cases with follow up, all the MCD cases were steroid sensitive. Eight of the twelve (66.6%) FSGS cases with follow up were steroid sensitive, three (25%) were steroid resistant and one was steroid dependent.

Post Graduate Institute of Medical Education and Research, Chandigarh, India had approved this study (reference no 7545-PG-Ttrg/2009 dated 26.11.2009).

### Statistics

The statistical analysis was carried out using Statistical Package for Social Sciences (SPSS Inc., Chicago, IL, version 15.0 for Windows). For non-normal variables Mann-Whitney test (for variables with two groups) or Kruskal Walis test (for variables with more than two groups) were applied. Qualitative or categorical variables were described as frequencies and proportions. Proportions were compared using chi square test wherever applicable. All statistical tests were two-sided and performed at a significance level of α = 0.05

## Results

Patients were categorized as with nephrotic or subnephrotic range protienuria with or without nephritic features. Nephritic presentation was most frequent in IgAN cases. There was no significant difference in the clinical and laboratory parameters in the other categories. The clinical and laboratory parameters at the time of diagnosis are summarized in the Tables 1 and 2.

**Table 1:** Summary of clinical and laboratory findings

		**MCD**			**FSGS**			**IgAN**			**MGN**			**P value**
		**Mean ± SD**	**Min**	**Max**	**Mean ± SD**	**Min**	**Max**	**Mean ± SD**	**Min**	**Max**	**Mean ± SD**	**Min**	**Max**	-
**Age (years)**		30±10.98	14	60	34±15.93	14	72	28±9.83	14	45	41±13.63	20	62	
**Sex**	**Male**	12			12			11			16			
	**Female**	10			9			9			9			
**24 hour urine protein (gms/24hours)**		3.44±1.56	0.40	6.80	3.52±2.05	0.64	10.00	3.40±1.60	0.70	5.00	3.51±1.19	1.80	6.00	0.985
**Serum creatinine (mg/dl)**		1.19±0.54	0.40	2.60	1.41±0.61	0.50	2.30	1.43±1.46	0.57	3.20	1.17±0.68	0.50	3.20	0.611
**Hypertension**	**Non Hypertensive**	18			16			10			17			0.519
	**Hypertensive**	4			5			7			5			
**Proteinuric range**	**Nephrotic (24 hours urine protein > 3gms)**	17			20			2			23			<0.001*
	**Sub nephrotic (24hours urine protein 0.5 to 3 gms)**	4			0			3			0			
	**Nephritic (hematuria + proteinuria)**	1			1			15			2			
**Steroid responsiveness**	**Steroid sensitive**	10			8			-			-			-
	**Steroid resistant**	0			3			-			-			
	**Steroid dependent**	0			1			-			-			

Note; MCD-Minimal change disease, FSGS-focal segmental glomerulosclerosis, IgA N-IgA nephropathy, MGN-Membranous glomerulonephritis, min-minimum, max –maximum, SD –standard deviation, mg-miligram.gm-gram, dl-deciliter, *-statistically significant.

**Table 2:** Significant additional demographic information of patients

	**MCD**	**FSGS**	**IgAN**	**MGN**
**Diabetes**	0	2	0	1
**Malformations**	0	0	0	0
**Infections**	0	0	2 Pustules Hepatitis C	0
**Profession**	0	Factory worker	Student -3	0
**Exposure**	0	Asbestosis	0	0
**Alternative medicine**	1	2	0	1

Note; MCD-Minimal change disease, FSGS-focal segmental glomerulosclerosis, IgA N-IgA nephropathy, MGN-Membranous glomerulonephritis.

### Immunohistochemistry for beta dystroglycan

There was intense (3+) to moderate (2+) staining of the podocytes along glomerular basement membranes in all cases of FSGS, IgAN and MGN. Tubular basement membranes and control cases served as internal and external controls respectively. There was no significant difference in the staining intensity between different groups (p-0.172). There was loss of immunostain for beta dystroglycan in areas of segmental/global sclerosis in various groups. There was intense(3+) to moderate(2+) staining of the podocytes along glomerular basement membranes in all cases of MCD also except that 3 cases had 1+ staining (which is considered as weak). Table 3, Figure 1.

Cases of MCD with loss of staining were not different from other patients; there was no correlation between the β-dystroglycan staining intensity and degree of proteinuria (1.80, 4.20, 5.00gm/24 hours, *p-*0.638), or renal function assessed by the serum creatinine levels(0.8, 0.9, 1.10 mg/dl, *p-*0.491). Follow up history was available in these cases and were steroid sensitive. Immunohistochemistry for podocin was positive (2 + -3+) in these three cases.

Tubular basement membrane staining was variable between groups, both in terms of overall number of tubules stained and the intensity of proximal convoluted tubules (PCT) and distal convoluted tubules (DCT) staining; these differences were statistically significant (p <0.001 and, 0.009 respectively). Table 2 Overall number of tubules stained (more than two third) with beta dystroglycan were more (half to two third cases) in MGN, IgAN, FSGS as compared to MCD (one fourth). Tubules with intense staining in MCD were PCT as compared to other groups where intense staining was localized to DCT (Table 3).

**Table 3:** Staining pattern of glomeruli and tubules with beta dystroglycan in MCD, FSGS, IGAN and MGN

**Histological parameters**	**Degree**	**Groups**					**P value**
			**MCD**	**FSGS**	**IgAN**	**MGN**	
**Glomerular staining of**	Absent	n(%)	4(18.1%)	4(19.0%)	4(20.0%)	1(4.0%)	**0.975 NS**
**beta-dystroglycan**	Mild	n(%)	4(18.1%)	3(14.2%)	6(30.0%)	4(16.0%)	
	Moderate	n(%)	10(45.4%)	10(47.6%)	7(35.0%)	7(28.0%)	
	Intense	n(%)	4(18.1%)	4(19.0%)	3(15.0%)	13(52.0%)	
**Tubules (PCT) staining pattern of**	Absent	n(%)	8(36.3%)	5(23.8%)	15(75.00%	11(44.0%)	0.009 *
**beta dystroglycan**	Mild	n(%)	0	2(9.5%)	0	3(12.0%)	
	Moderate	n(%)	4(18.1%)	9(42.8%)	5(25.00%)	8(32.0%)	
	Intense	n(%)	10(45.4%)	5(23.8%)	0	3(12.0%)	
**Tubules (DCT) staining pattern of**	Absent	n(%)	2(9.0%)	2(9.5)	7(35.0%)	3(12.0%)	**0.92 NS**
**beta dystroglycan**	Mild	n(%)	0	0	0	2(8.0%)	
	Moderate	n(%)	6(27.2%)	5(23.8%)	7(35.0%)	7(28.0%)	
	Intense	n(%)	14(63.6%)	14(66.6%)	6(30.0%)	13(52.0%)	
**% Tubular staining**	<30%	n(%)	6(27.27%)	5(23.8%)	10(50.0%)	6(24.0%)	<0.001*
	30-60%	n(%)	10(45.4%)	6(28.5%)	0	3(12.0%)	
	>60%	n(%)	6(27.2%)	10(47.6%)	10(50.0%)	16(64.0%)	

Note; MCD-Minimal change disease, FSGS-focal segmental glomerulosclerosis, IgA N-IgA nephropathy, MGN-Membranous glomerulonephritis, NS-not significant, PCT-proximal convoluted tubules, DCT-distal convoluted tubules, *- statistically significant.

### Immunohistochemistry for podocin

There was intense(3+) to moderate(2+) staining of the podocytes with podocin along glomerular basement membranes in all cases of MCD, FSGS, IgAN and membranous glomerulopathy. Control cases served as external controls with each batch of stain. There was no cases with negative staining or weak(1+) staining in any of other groups. There was loss of immunostain for podocin in areas of segmental/global sclerosis in various groups. There was no significant difference in the staining pattern between the groups (*p* value 0.5). There was no difference in the staining pattern of the steroid resistant, steroid sensitive or steroid dependent cases (*p-*0.45). There was no correlation between the podocin staining intensity and 24 hours urine protein levels (*p-*0.35) Figure 2.

## Discussion

Podocytopathies in adults and pediatric patients are different aetiopathogenetically and have different rate of disease progression and response to therapy [1,2]. In present study we did not find any difference in expression of beta dystroglycan in the four groups of patients presenting as protienuric states. Diseases with primary podocytopathies (MCD and FSGS) and cases with secondary podocytopathies due to sub epithelial immune complex formation (MGN) and mesangial immune complexes (IgAN) were not different in terms of expression of beta dystroglycan which is one of the integrins involved in podocyte basement membrane interaction. Expression of β dystroglycan, the adhesion molecule between podocyte and basement membrane in diseases with podocyturia (FSGS, membranous glomerulonephritis, IgAN) was not different from disease without podocyturia (MCD. In the present study, only three out of the 22 cases of minimal change disease showed reduced intensity of beta dystroglycan expression and were steroid sensitive as were cases with intense dystroglycan staining.

Regale et al [5] observed reduced expression of β dystroglycan in steroid responsive minimal change disease, which was also substantiated with immunogold labeling index. Their study had the limitation of few numbers of cases and included both adult and pediatric patients. Giannico et al in a study of adult cohort documented no reduction in expression of beta dystroglycan in MCD, whereas alpha dystroglycan was reduced in MCD [7]. However, Vogtlander et al documented that there was no difference in the staining pattern of alpha dystroglycan in nephrotic syndrome cases [6] (Table 4).

**Table 4:** Comparison of beta dystroglycan immunostaining with other studies

**Authors**	**β dystroglycan staining**	**MCD**	**FSGS**	**IgAN**	**MGN**
**Regale et al (2000) **[5]**Adults and pediatrics**	Total cases	12	8	Nil	Nil
	Cases with +/- score	5	Nil	N.A	N.A
	No of case with 1+ glomerular score	7	1	N.A	N.A
	Cases with 2+/3+ score	Nil	4 and 3	N.A	N.A
**Giannico et al (2009)**[9]**Adults**	Total cases	10	32	N.A	N.A
	Cases with absent staining	Nil	Nil	N.A	N.A
**Present studyAdults**	Total cases	22	21	20	25
	Cases with 1+ score	3	Nil	Nil	Nil
	Cases with 2+ and 3+ score in the glomeruli	2 and 17	1 and 20	3 and 17	6 and 19

Note: MCD-Minimal change disease, FSGS-focal segmental glomerulosclerosis, IgA N-IgA nephropathy, MGN-Membranous glomerulonephritis.

N.A- not available.

Schmid et al [8] studied mRNA expression in adult population, and documented that the ratio of beta dystroglycan mRNA/house keeper gene ratio was not altered in MCD. They concluded that beta dystroglycan gene expression may not help to differentiate MCD from FSGS (Table 3).

In present study, observation of variable intensity and number of tubular staining with beta dystroglycan on immunohistochemical stain suggests that use of methodology like mRNA expression without taking into consideration expression pattern on histology might not give correct information about alteration of widely and variably expressed protein like beta dystroglycan.

Similarly, there was no difference in immunohistochemical expression of podocin in these groups of diseases i.e. primary podocytopathies (MCD and FSGS) and cases with secondary podocytopathies MGN and IgAN. No significant difference was observed in the Podocin staining pattern in any of the groups. There have been variable results documented in literature which could be due to difference in the composition of study cohorts(pediatric patients or adults or both) or the techniques used (Table 5).

**Table 5:** Comparison of podocin immunostain with other studies

**Authors**	**No. of cases**	**MCD**	**FSGS**	**IgAN**	**MGN**	**Henoch Schoenlein purpura nephritis**
**Horinouchi et al (2003) **[10]**Pediatric and adult**	Total	6	19	6	5	6
	Absent staining	0	7(36.84%)	0	0	0
	1+	0	7(36.84%)	0	1(15%)	0
	2+	0	4(21.05%)	0	19(15%)	0
	3+	6(100%)	1(5.2%)	6(100%)	3(60%)	6(100%)
**Mao J et al (2006) **[11]**Pediatric**	Total	9	-	6	-	-
	Podocin fluorescence intensity	Not reduced		Not reduced		
**Koop et al (2003) **[6]**Adult**	Total	10	5	10	1	
	Podocin IHC intensity quantification by image analysis	Reduced	Reduced	Not altered	Reduced	-
	Podocin mRNA levels	Increased	Increased	Decreased	Increased	-
**Guan N et al (2002) **[12]**Pediatric**	Total	9	-	4	-	-
	Podocin staining intensity	Decreased	Not decreased	Not decreased	-	-
**Present study**	Total	22	21	20	25	-
	Absent staining	0	0	0	0	-
	1+	0	0	0	0	-
	2+	6(27.27%)	7(33.33%)	3(15%)	8(32%)	-
	3+	16(72.72%)	14(66.66%)	17(85%)	17(68%)	-

Note; MCD-Minimal change disease, FSGS-focal segmental glomerulosclerosis, IgA N-IgA nephropathy, MGN-Membranous glomerulonephritis.

Comparison between these studies is difficult because most of the studies (except adult cohort by Koop et al [9], included either only pediatric cases or included both pediatric and adult patients. Techniques and methods of analysis were also variable. These diseases are known to be different aetiopathologicaly, in rate of progression and response to therapy in adults and pediatrics; hence we chose to study only adult patients.

Horinouchi et al [10] (both pediatric and adult population) observed reduced expression of podocin in FSGS cases except for only one case which had 3+ staining for podocin; this case with positive staining showed good response to treatment as was observed in present study 10. They suggested that decreased podocin expression in FSGS cases may be associated with steroid resistance and poor response to therapy. They also documented decreased podocin staining in MGN and normal staining pattern being preserved in IgAN and HSP (due to mesangial nature of the disease). It was proposed that the mesangial diseases are less prone for the loss of podocyte associated proteins in the glomeruli, compared to FSGS where the primary defect occurs in the podocytes and more prone for loss of podocyte associated protein. However, this does not explain the loss of podocin expression being more in MGN where the glomerular injury occurs at the sub epithelial area, compared to MCD, where the podocytes are the primary targets of injury [10] (Table 5).

Mao et al [11] have reported that the expression level of podocin is not reduced in the minimal change disease and IgAN (pediatric population). Koop et al 9 documented reduced expression of podocin and nephrin in protienuric states i.e. MCD, FSGS, MGN except IgAN adult cohort). Guan et al [12] (pediatric) also recorded reduced expression of podocin in MCD only and no alteration in FSGS and IgAN.

In present study, podocin expression was observed on smooth muscles of arteries included in the biopsies, which might have confounded the results where mRNA expression of whole kidney biopsy was assessed.

Various studies aimed at studying the expression of the podocyte associated proteins at the mRNA level or the protein level have shown inconsistent results in the expression of these proteins in acquired nephrotic syndrome. Similar to the differences observed in the mutational status of pediatric and adult population, there might be differences in the protein expression patterns of pediatric and adult patients.

## Conclusion

Present study is a cohort of only adult patients comprising of relatively large number of cases and emphasize that there is no difference in expression of podocin and beta dystroglycan involved in podocin-podocin interaction and podocyte - basement membrane and in cases of both primary and secondary podocytopathies (immune complex mediated). These immunostains cannot be used to differentiate between MCD/FSGS and predict response to therapy at least in adult cases unlike documented by few studies in literature. There is no loss of staining in steroid resistant cases.

The molecular mechanisms underlying the pathogenesis of pediatric and adult nephrotic syndrome may be markedly different. Further, molecular and immunohistochemical studies on both beta dystroglycan and podocin may help us to understand the role of these proteins in the pathogenesis of primary and secondary podocytopathies and to understand how to apply these markers in the routine management of patients.

## Abbreviations

FSGS: Focal segmental glomerulosclerosis; IgAN: IgA nephropathy; MGN: Membranous glomerulonephritis; MCD: Minimal change disease.

## Competing interests

The authors declare that they have no competing interests.

## Authors' contributions

Various co authors have significantly contributed to the publication as mentioned against their names. PBS; participated in designing study, drafting the article, staining slides, collecting data. RN; participated in designing study ,drafting the article, providing intellectual content of critical importance to the work described, interpreting staining slides and clinical data. AK; participated staining slides, collecting data. CSR; participated in doing Electron microscopy. VS; participated by providing intellectual content of critical importance to the work described and providing clinical data, final approval of the version to be published. KJ; participated in designing study, drafting the article, providing intellectual content of critical importance to the work described, interpreting staining slides and clinical data, final approval of the version to be published. All authors read and approved the final manuscript.
